# Circadian rhythm-associated clinical relevance and Tumor Microenvironment of Non-small Cell Lung Cancer

**DOI:** 10.7150/jca.52454

**Published:** 2021-03-05

**Authors:** Ming Li, Zhencong Chen, Tian Jiang, Xiaodong Yang, Yajing Du, Jiaqi Liang, Lin Wang, Junjie Xi, Miao Lin, Mingxiang Feng

**Affiliations:** 1 Department of Thoracic Surgery, Zhongshan Hospital, Fudan University, Shanghai, 200032, People's Republic of China.; 2 Department of Thoracic Surgery, Shanghai Pulmonary Hospital, Tongji University, Shanghai, 200433, People's Republic of China.; 3 Center for Tumor Diagnosis and Therapy, Jinshan Hospital, Fudan University, Shanghai 201508, People's Republic of China.

**Keywords:** circadian rhythm, non-small cell lung cancer, signature, prognosis, cellular function, immune infiltration

## Abstract

**Objective:** We aimed to explore the prognostic implication for non-small cell lung cancer (NSCLC) based on the expression profiles of circadian clock-related genes (CCRGs), and describe the changes of immune infiltration and cell functions of related to the circadian rhythm.

**Methods:** Univariate and multivariate Cox proportional hazard regression were performed to determine a CCRGs risk-score significantly correlated with overall survival (OS) of the training set and validation set. GO, KEGG, and GSVA indicated discrepant changes in cellular processes and signaling pathways associated with these CCRGs. Immune cell infiltration and mutation rates were investigated by the online analysis platform and the algorithm provided by works of literature.

**Results:** The signature-based on ten-gene signatures could independently predict the OS both in TCGA lung adenocarcinoma (*p* < 0.001, HR: 1.228, 95% CI: 1.158 to 1.302) and lung squamous cell carcinoma (*p* < 0.001, HR: 2.501, 95% CI: 2.010 to 3.117), respectively. The circadian oscillations driven by CCRGs could disturb the metabolism and cellular functions of cancer cells. The infiltration level of critical cells in specific anti-tumor immunity process was suppressed apparently. In contrast, the infiltrating of inflammatory cells and immune cells with negative regulatory effects were promoted in the high-risk group. CCRGs were evolutionarily conserved with low mutation rates, which brought difficulties to explore therapeutic targets.

**Conclusion:** We identified and validated a circadian rhythm signature to described clinical relevance and tumor microenvironment of NSCLC, which revealed that circadian rhythms might play an influential role in the NSCLC.

## Introduction

The expression of circadian clock genes drives oscillatory changes in innumerable behavioral and physiological processes, including tumorigenesis and development 1,2. According to the organism and cell type, the circadian clock promotes the rhythmic expression of 1% to over 60% of the genome, serving as the molecular basis for rhythmic control at the system's level 3. In recent years, it is becoming an increasing focus of the role of the circadian clock in tumorigenesis, cancer hallmarks, therapeutic options, and discussions of how circadian clock genes can lead a new dimension in future medicine 4,5. Accumulating evidence identified that there was a tight association between cancer and disruption of circadian in curative effect and prognosis, and the core circadian transcripts are generally altered in many kinds of cancers 6-8. Nevertheless, the regulatory mechanism of circadian clock genes and their effects on clinical prognosis is not precise yet.

Lung cancer is the most leading cause of cancer death (18.4% of the total cancer deaths) with the highest incidence (11.6% of the total cases) around the world 9. Non-small cell lung cancer (NSCLC) is the most common type and accounts for about 85% of total cases 10. Previous studies have shown that the dysregulation of the circadian rhythms can influence cancer development by regulating tumor cell apoptosis, immune infiltration, and tumor cell-host interactions via related genes oscillatory and differential expression, which has been studied in many kinds of cancers including breast, colorectal cancer, and head and neck squamous cell carcinoma 11-13. However, clinical relevance of differentially expressed circadian clock-related genes (CCRGs) in lung cancer have remained poorly defined. Studies have demonstrated that both physiologic perturbation and genetic mutation of the central circadian clock components decreased survival and promoted lung cancer growth and progression. Such as the core circadian genes Per2 and Bmal1 were shown to have cell-autonomous tumor-suppressive roles in transformation and lung tumor progression 14. However, there is still a lack of a multi gene set to describe the prognosis changes caused by the physiologic perturbation and genetic mutation of CCRGs.

In this present study, we purposed to explore a risk-score as a “Classifier” to predict the prognosis of patients based on the genomic expression profiles from public databases. Comprehensively, a total of 1,382 CCRGs with oscillatory transcripts with experimentally validated by techniques including RT-PCR, Northern blot, *in situ* hybridization, and Microarray or RNA-seq were analyzed 15. Among them, we identified 290 and 447 differentially expressed CCRGs in lung adenocarcinoma (LUAD) and lung squamous cell carcinoma (LUSC) tissues, respectively based on the Cancer Genome of Atlas (TCGA) cohort. Then, a combination of univariate and Cox regression hazard regression analysis was used to screen the differential expression of CCRGs, which associated with overall survival. Subsequently, we established two optimal risk-score models to divide LUAD and LUSC patients into the high or low risk-score group, followed by verification in combined Gene Expression Omnibus (GEO) validation sets, respectively.

Further, we assessed the prognosis value and complementary value of molecular and clinical characteristics by survival, receiver operating characteristic (ROC) curve, and correlation analysis. We also identified the differences in the critical signaling pathways among these differential expression CCRGs using Gene Ontology (GO), the Kyoto Encyclopedia of Genes and Genomes (KEGG), and Gene Set Variation Analysis (GSVA) methods. Finally, the correlative immune infiltrates and genetic alteration of CCRGs in risk-score models were explored to provide novel ideas for the clinical translation of circadian genes.

## Materials and Methods

### Patient information and databases in training and validation sets

Thoroughly, a list of 1,382 homo sapiens (human) CCRGs validated by experiments including RT-PCR, Northern blot, and *in situ* hybridization were obtained from the Circadian Gene Database (The CGDB: http://cgdb.biocuckoo.org/index.php/) 15. In the training set, both the gene expression profiles (HTSeq - FPKM) and patient clinical information of 594 LUAD samples (normal count: 59; tumor count: 535) and 551 LUSC samples (normal count: 49; tumor count: 502) were downloaded from the TCGA database (https://portal.gdc.cancer.gov/). Patients who lacked follow-up information were excluded in the survival analysis. In the testing set, Microarray expression profiles and clinical information were obtained from the GEO database (ncbi.nlm.nih.gov/geo/) using the accession number GSE30219, GSE31210, GES3141, GSE37745, GSE50081, GSE68465, which contained more than a thousand samples of patients with lung cancer^16^-^19^. Then, all samples were classified into the LUAD or LUSC type due to histological criteria to verify the signature, respectively.

### Data processing

To avoid the heterogeneity among different datasets and ensure a unified standard, the RNA-seq profiles were transformed using the formula log2(x+1) and normalized. R version 3.6.1 (https://www.r-project.org/) software was used to normalize and process the data. Also, all data processing, analysis, and mapping were done using the R version 3.6.1 software and the Perl Programming Language version 5.28.1 (https://www.perl.org/) in the present study.

### Functional enrichment analysis

To explore the pathways and interactions that are affected among these differential expression CCRGs, the statistical and visualize analysis of functional annotation (GO), including biological process, cellular component, and molecular function, and the KEGG pathway enrichment analysis and visualization were performed by using the R package “clusterProfiler” (http://www.bioconductor.org/). Finally, we also introduce the GSVA, a gene set enrichment method, to estimate the variation of pathway activity between the high risk-score and low risk-score groups in an unsupervised manner 20.

### Risk-score model construction

We performed the univariate analysis and Cox proportional hazard regression to conduct to screen the differential expression CCRGs significantly associated with prognosis in the training cohort. Then, a risk score for each patient of prognostic risk was calculated respectively in LUAD and LUSC sets, according to the regression coefficients of the individual CCRGs screened from the multivariate Cox regression model and the expression value of each of the selected CCRGs. The computational formula used for this analysis was risk-score = h_0_(t)*exp(β_1_X_1_+β_1_X_1_+…+β_n_X_n_). β refers to the regression coefficient, and a hazard ratio (HR) value can be obtained after taking the natural logarithm exp (β). H_0_(t) is the function of a benchmark risk, and h (t, X) is a risk function associated with X (covariant quantity) at time t. X (covariant quantity) represented the relative expression profiles of every CCRGs, which standardized by z-score. After modeling by multivariate Cox regression, the value of the risk score calculated by function “predict ()” is h (t, X). Then, all patients were into a high or low-risk group according to the median value as a cutoff to separately dichotomize the training sets in LUAD and LUSC, and a low-risk score indicates a superior prognosis for patients. This similar approach has been identified from previous studies 21,22. Finally, the risk-score signature of prognosis was verified in the testing sets, which integrated from the GEO cohorts (GSE30219, GSE31210, GSE3141, GSE37745, GSE50081, GSE68465).

### Immune infiltration analysis

We compared the difference of immune cell infiltration between the high-risk group and low-risk group by using the CIBERSORTx (https://cibersortx.stanford.edu/). CIBERSORTx is an extension of the CIBERSORT, which provides an analytical method to infer cell-type-specific gene expression profiles without digital cytometry 23. By the transcriptome profiling of single cells or sorted cell subpopulations based on a machine learning method, CIBERSORTx provides new possibilities for applying the signature matrix to bulk tissue expression profiles to infer cell-type proportions and represent cell type expression signatures. Based on this, we provided an analytical estimation of the abundances and distribution difference of 22 immune cell types in a mixed cell population of high-risk and low-risk group samples, using CCGRs expression data in LUAD and LUSC respectively. During the analysis, gene expression was corrected by the normalization using the R package “limma”, and samples after the abundance estimation were filtered with the *p*-valve (<0.05). When within-group data were merged, we combined the data by global averages.

### Mutation analysis of CCRGs in risk-score model

The cBioPortal for Cancer Genomics (http://cbioportal.org) provides a visualized Web resource for exploring and analyzing multidimensional cancer genomics data, which is now developed and maintained by a multi-institutional team, such as the Memorial Sloan Kettering Cancer Center, the Dana Farber Cancer Institute, Princess Margaret Cancer Centre of Toronto, Children's Hospital of Philadelphia. In this portal, molecular profiling data of cancer tissues and cell lines were reduced into readily visual and understandable genetic, epigenetic, gene expression, proteomic events, and clinically relevant events. According to the cBioPortal, we investigate mutations and expressions of CCRGs in the risk-score model in lung adenocarcinoma (TCGA PanCancer Atlas) (566 samples) and lung squamous Cell Carcinoma (TCGA PanCancer Atlas) (487 samples) respectively.

### Statistical analysis

All statistical analyzes in the present study were performed using the R version 3.6.1 software (https://www.r-project.org/), and *p*-value < 0.05 was regarded as statistically significant for all the analyses. The Kruskal-Wallis test and one-way ANOVA were used to check the expression differences among the genes and the association of the risk-score with clinical signatures. OS was analyzed by the Kaplan-Meier survival curve and the log-rank test to check the significant difference between the high-risk and low-risk groups. The univariate analysis and multivariate Cox proportional hazards regression model were used to analyze the key CCRGs that affect the prognosis of NSCLC patients. The ROC curve analysis was used to evaluate the sensitivity and specificity of prognostic prediction of the CCRGs signature risk-score model. The prognostic accuracy was presented by the area under the ROC curve (AUC). All tests were two-sided.

## Results

### Identification and screening of differential expression genes

**Figure 1:**
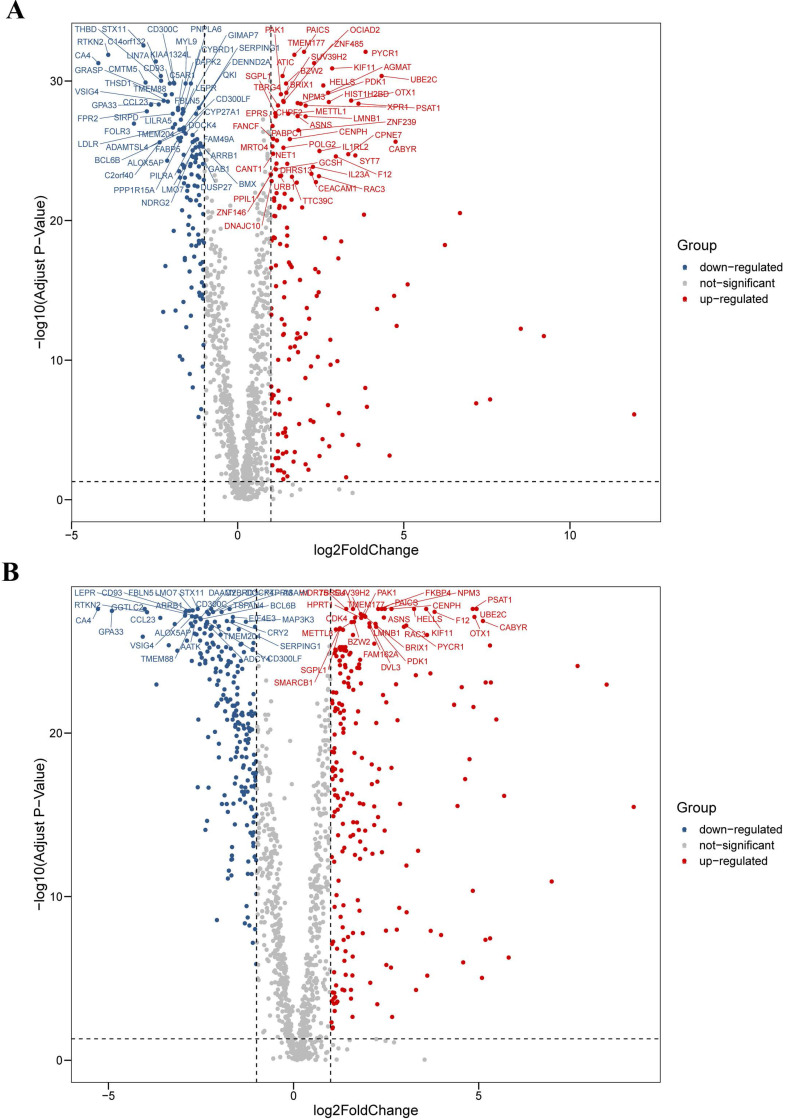
Differential expression profiles of 1,382 human CCRGs in the TCGA database. (A) Volcano plot of 1,382 human CCRGs in LUAD samples. (B) Volcano plot of 1,382 human CCRGs in LUSC samples. Totally, we identified 290 CCRGs (downregulated CCRGs: 126; upregulated CCRGs: 164), and 447 CCRGs (downregulated CCRGs: 226; upregulated CCRGs: 221) differentially expressed in LUAD and LUSC samples respectively.

We analyzed the differential expression profile of a total of 1,382 homo sapiens (human) CCRGs in 594 LUAD samples (normal count: 59; tumor count: 535) and 551 LUSC samples (normal count: 49; tumor count: 502) by the Wilcoxon signed-rank test respectively. Finally, we identified 290 CCRGs (downregulated CCRGs: 126; upregulated CCRGs: 164), and 447 CCRGs (downregulated CCRGs: 226; upregulated CCRGs: 221) differentially expressed in LUAD and LUSC samples respectively. Results of expression analyses are illustrated as volcano plots (Fig. 1).

### Functional enrichment analysis of differential expression genes

**Figure 2:**
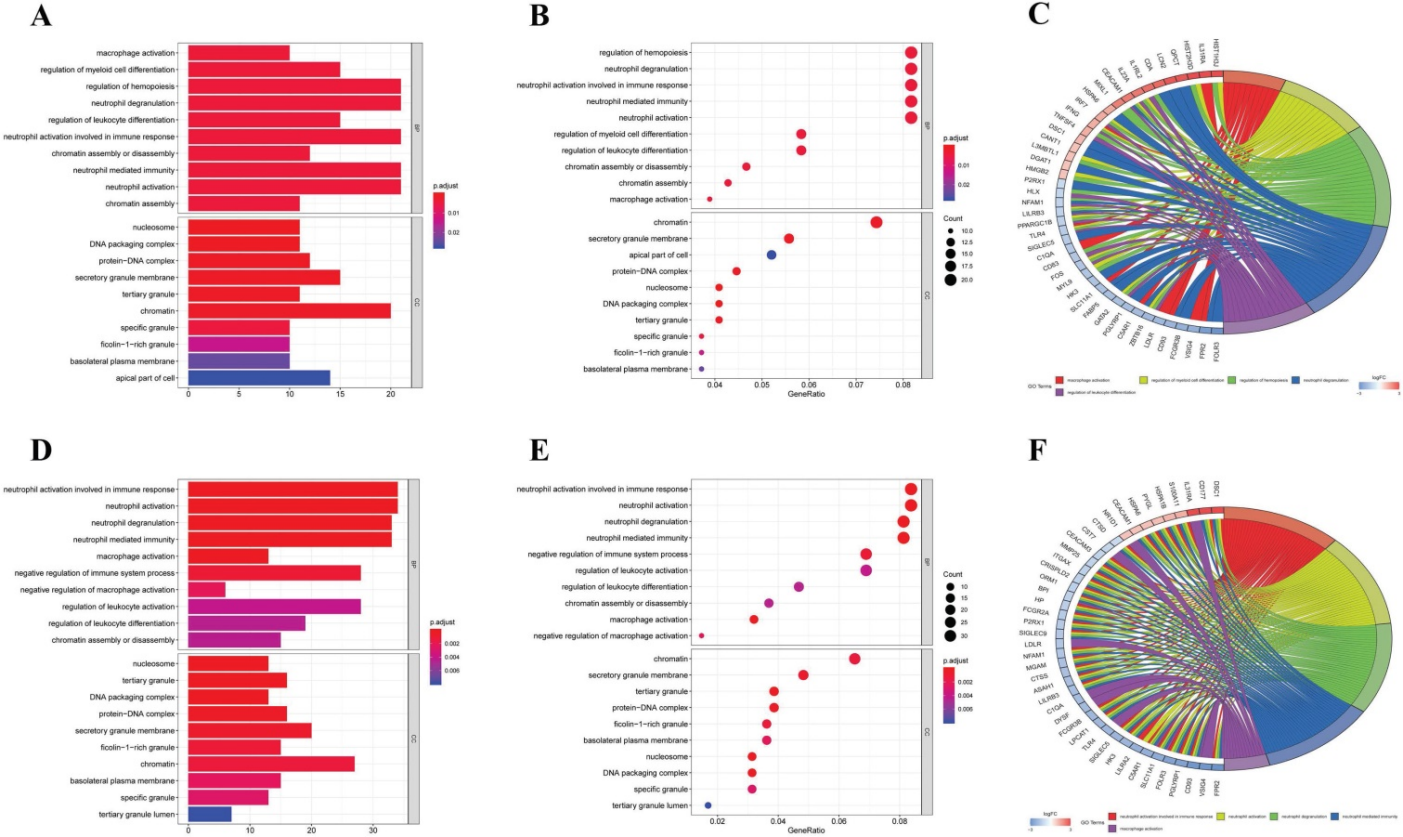
GO analysis of differentially expressed CCRGs. (A) Bar plot of GO analysis of differentially expressed CCRGs in LUAD. (B) Bubble chart of GO analysis of differentially expressed CCRGs in LUAD. (C) Circle diagram of GO analysis of differentially expressed CCRGs in LUAD. (D) Bar plot of GO analysis of differentially expressed CCRGs in LUSC. (E) Bubble chart of GO analysis of differentially expressed CCRGs in LUAD. (F) Circle diagram of GO analysis of differentially expressed CCRGs in LUSC. The results revealed distinct enriched CCRGs sets between different biological functions.

**Figure 3:**
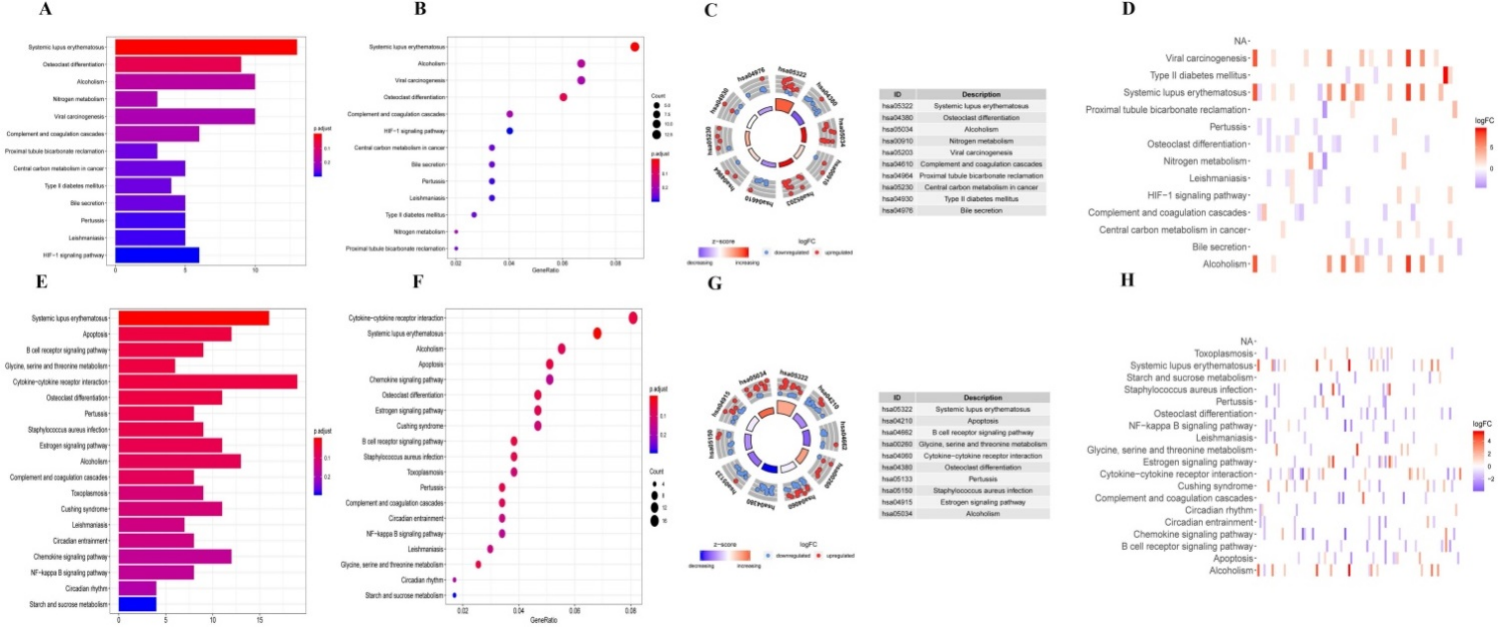
KEGG analysis of differentially expressed CCRGs. (A) Bar plot of KEGG analysis of differentially expressed CCRGs in LUAD. (B) Bubble chart of KEGG analysis of differentially expressed CCRGs in LUAD. (C) Circle diagram of KEGG analysis of differentially expressed CCRGs in LUAD. (D) Heatmap of KEGG analysis of differentially expressed CCRGs in LUAD. (E) Bar plot of KEGG analysis of differentially expressed CCRGs in LUSC. (F) Bubble chart of KEGG analysis of differentially expressed CCRGs in LUAD. (G) Circle diagram of KEGG analysis of differentially expressed CCRGs in LUSC. (H) Heatmap of KEGG analysis of differentially expressed CCRGs in LUSC.

Firstly, we analyzed the association of these differentially expressed CCRGs with the GO terms of the biological process (BP) and cellular component (CC) categories. For LUAD samples, the top five enriched BP terms were 'regulation of hemopoiesis,' 'neutrophil degranulation,' 'neutrophil activation involved in immune response', 'neutrophil-mediated immunity', and 'neutrophil activation' (Fig. 2A-C). The top two enriched CC terms were 'chromatin' and 'secretory granule membrane' (Fig. 2A-C). For LUSC samples, the top five enriched BP terms were 'neutrophil activation involved in immune response', 'neutrophil activation', 'neutrophil degranulation', 'neutrophil-mediated immunity', and 'negative regulation of immune system process', which was like the results of the former, while more inclined towards the immunomodulation (Fig. 2D-F). For the results of CC, the top two enriched terms were precisely the same as the former (Fig. 2D-F). Then, results of KEGG analysis indicated that altered CCRGs were mainly involved in the systemic lupus erythematosus and osteoclast differentiation of LUAD samples, meanwhile in the systemic lupus erythematosus, apoptosis, and B cell receptor signaling pathway of LUSC samples (Fig. 3).

**Figure 4:**
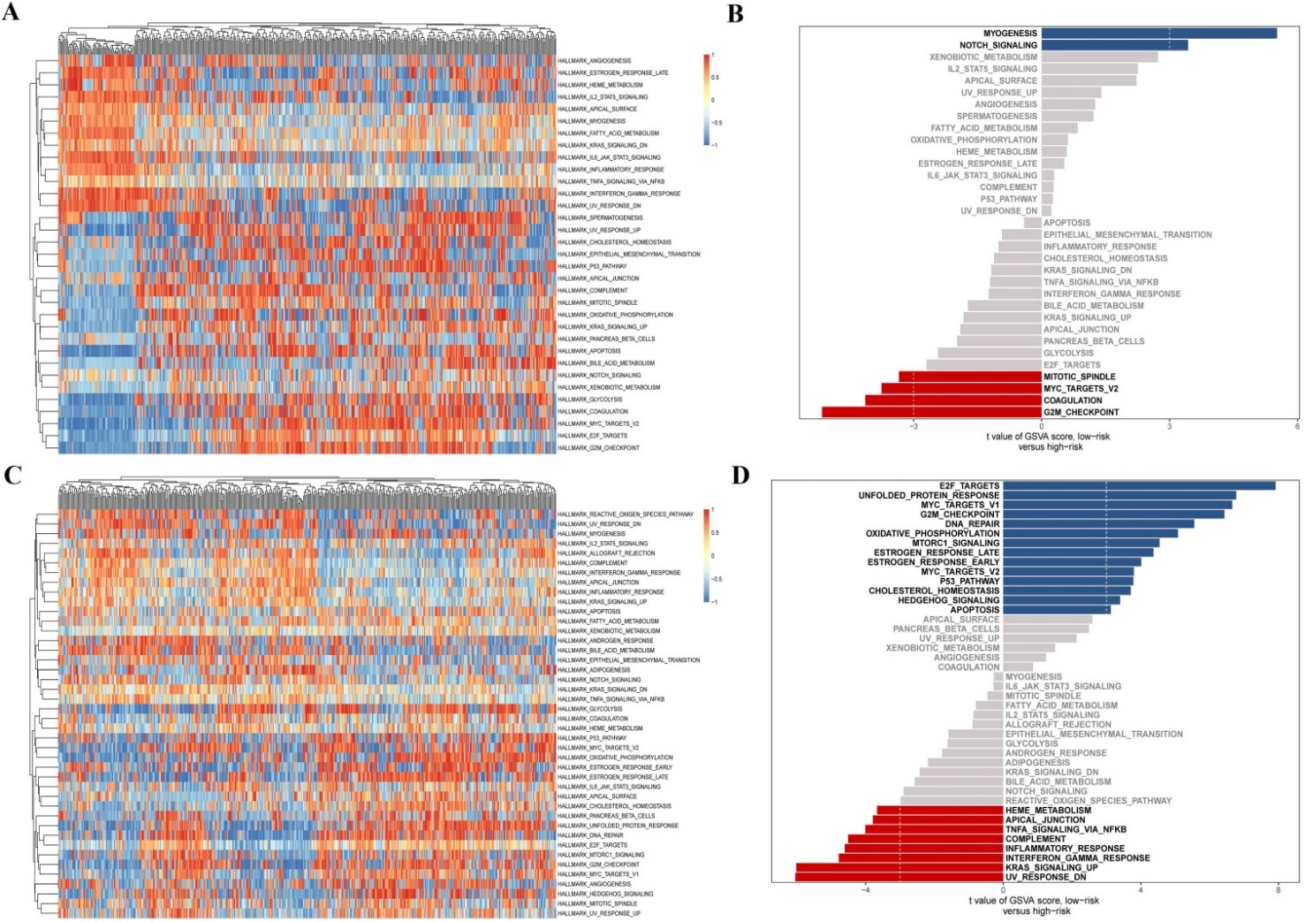
GSVA analysis of differentially expressed CCRGs. (A) Heatmap of GSVA analysis in LUAD samples. (B) Differential distribution of signal pathway enrichment between the high risk-score and low risk-score groups in LUAD samples. (C) Heatmap of GSVA analysis in LUSC samples. (D) Differential distribution of signal pathway enrichment between the high risk-score and low risk-score groups in LUSC samples.

Finally, we further investigated the differential distribution of signal pathway enrichment of differentially expressed CCRGs set between the high risk-score and low risk-score groups using the GSVA method. In LUAD samples, compared with the low-risk group, CCRGs of the high-risk group mainly enriched in the G2M_CHECKPOINT, COAGULATION, MYC_TARGETS_V2, and MITOTIC_SPINDLE. Meanwhile, CCRGs of the low-risk group mainly enriched in the MYOGENESIS and NOTCH_SIGNALING. However, the results of the LUSC samples are quite different. CCRGs of the high-risk group mainly enriched in the UV_RESPONSE_DN, KRAS_SIGNALING_UP, TNFA_SIGNALING_VIA_NFKB, INFLAMMATORY_RESPONSE, and INTERFERON_GAMMA_RESPONSE, while CCRGs were mainly involved in the E2F_TARGETS, UNFOLDED_PROTEIN_RESPONSE, MYC_TARGETS_V1, G2M_CHECKPOINT, and DNA_REPAIR in the low-risk group (Fig. 4). It indicated the differential expression and regulation of CCRGs in LUAD and LUSC. This difference may lead to different biological processes related to the circadian rhythm in cancer cells.

### Construction of the prognostic risk-score model in the training set

**Figure 5:**
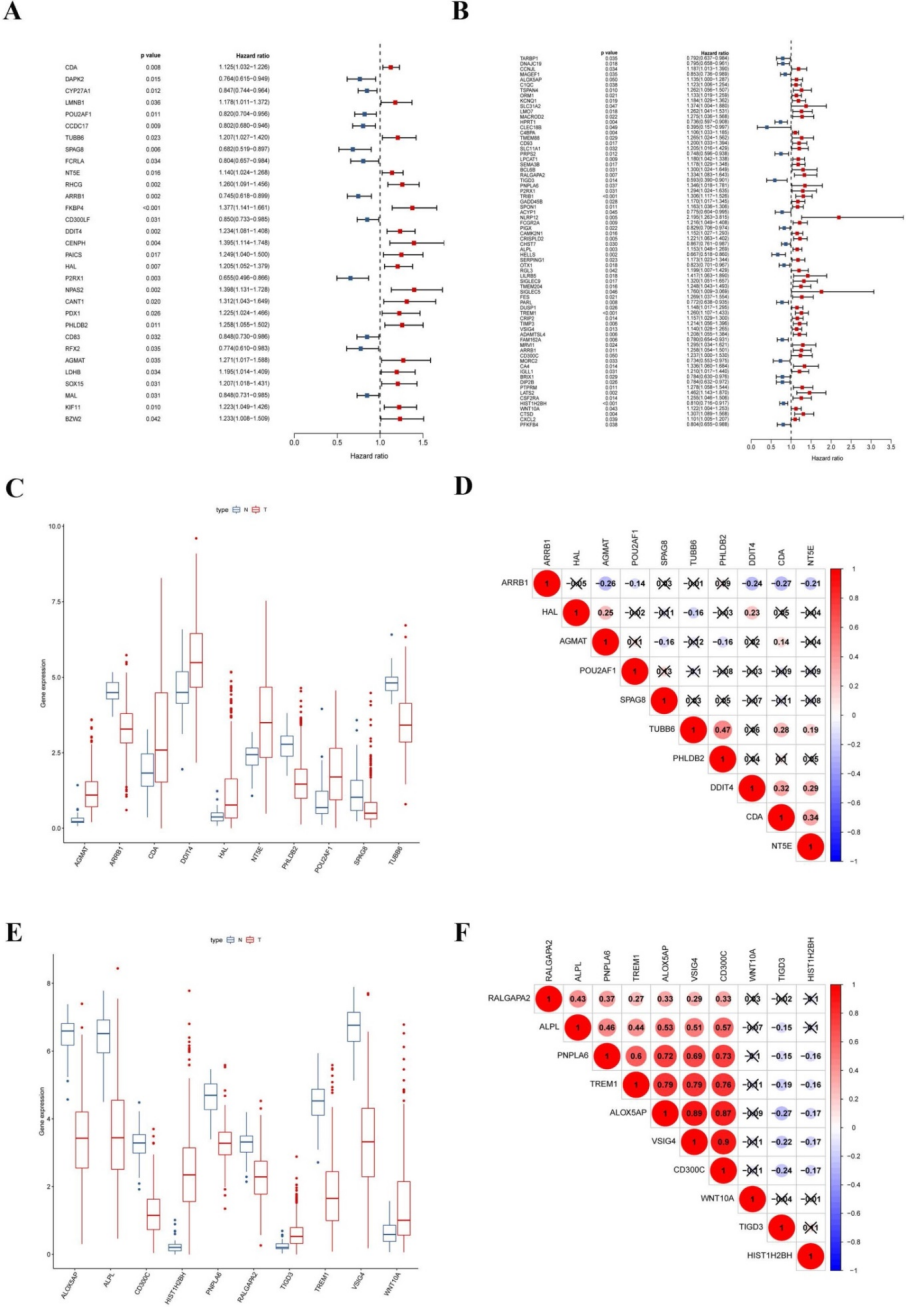
Signatures of ten CCRGs in the risk-score model. (A) The expression of 31 CCRGs each significantly correlated with the OS of LUAD patients in the univariate analysis. (B) The expression of 70 CCRGs each significantly correlated with the OS of LUSC patients in the univariate analysis. (C) Boxplot of differentially expressed CCRGs of the risk-score model (LUAD samples). (D) Correlation between ten CCRGs in the risk-score model of LUAD. (E) Boxplot of differentially expressed CCRGs of the risk-score model (LUSC samples). (F) Correlation between ten CCRGs in the risk-score model of LUSC.

**Figure 6:**
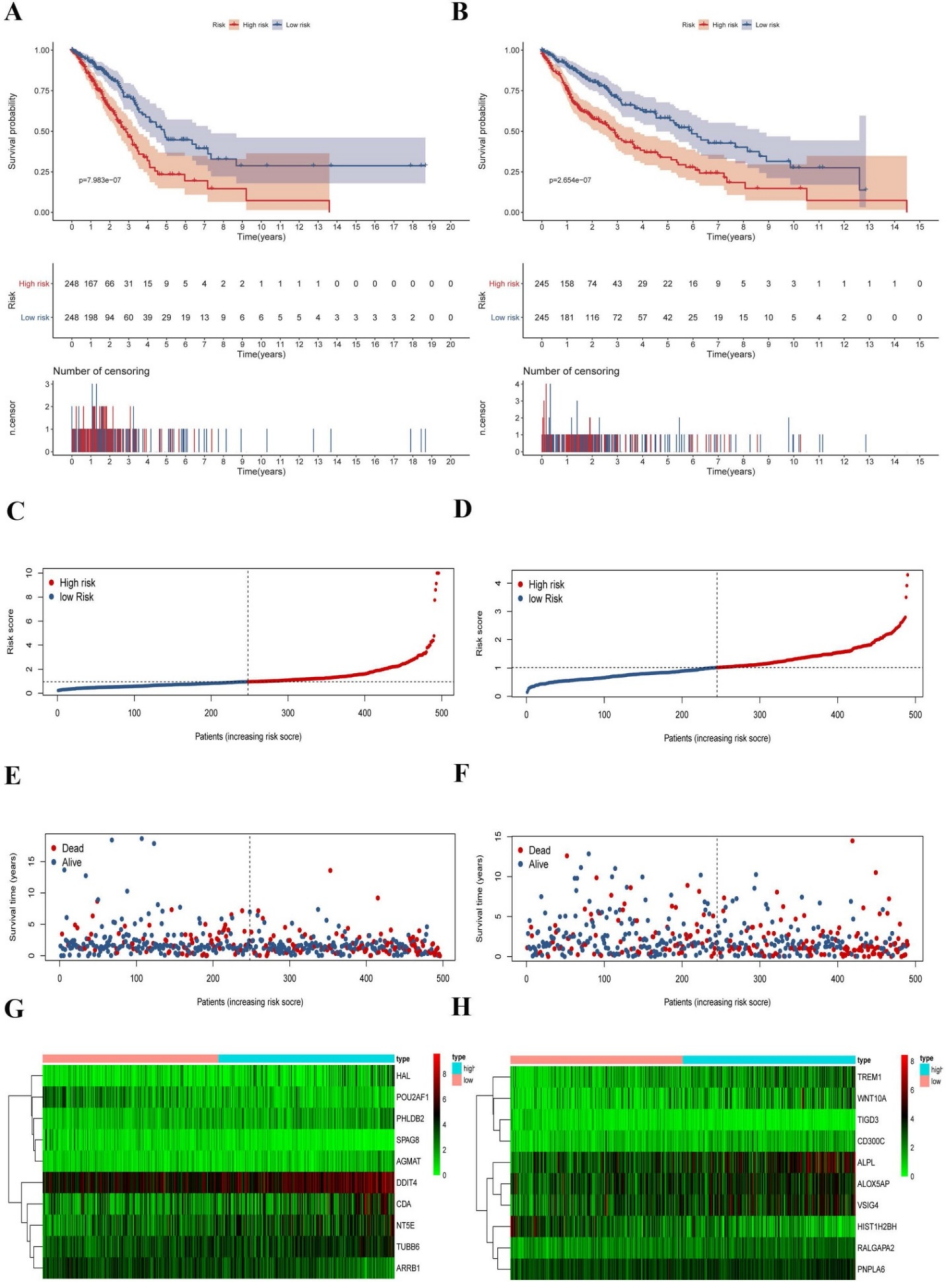
Survival analysis and characteristics of the prognostic gene signature. (A) Kaplan-Meier analysis of LUAD patients stratified by the risk-score in the training set. (B) Kaplan-Meier analysis of LUSC patients stratified by the risk-score in the training set. (C) The distribution of risk-score for LUAD patients in the training set. (D) The distribution of risk-score for LUSC patients in the training set. (E) Survival time and status for LUAD patients in the training set. (F) Survival time and status for LUSC patients in the training set. (G) Heatmap of CCRGs expression profiles in prognostic signature for LUAD patients in the training set. (H) Heatmap of CCRGs expression profiles in prognostic signature for LUSC patients in the training set.

To explore the prognostic signatures of these differentially expressed CCRGs, we preliminarily performed a univariate analysis of the standardized expression of the 290 CCRGs of LUAD and 447 CCRGs of LUSC in the training sets to identify the prognostic CCRGs respectively. The results showed that the expression of 31 CCRGs and 70 CCRGs each significantly correlated with the OS (p < 0.05) of LUAD patients and LUSC, respectively (Fig. 5A-B). Further, we performed Cox proportional hazard regression to screen the ultimate CCRGs of risk-score models. Subsequently, results indicated that the expression of 10 CCRGs correlated with the OS of LUAD and LUSC patients, respectively (Table 1). According to this, we constructed a risk-score model for predicting the prognosis of LUAD and LUSC patients using the calculation formula mentioned in the method part, respectively. Finally, CDA, POU2AF1, TUBB6, SPAG8, NT5E, ARRB1, DDIT4, HAL, PHLDB2, and AGMAT as risk genes in the risk-score model of LUAD and ALOX5AP, RALGAPA2, TIGD3, PNPLA6, ALPL, TREM1, VSIG4, CD300C, HIST1H2BH, and WNT10A as risk genes in the LUSC risk-score model (Fig. 5C-F). Survival analysis revealed that there was a significant difference between the high-risk group and the low-risk group in OS, and patients in the high-risk group significantly correlated with an inferior prognosis (LUAD: p < 0.0001, HR: 2.117, 95% CI: 1.546 to 2.900; LUSC: p < 0.0001, HR: 2.066, 95% CI: 1.552 to 2.751) (Fig. 6A-B). Finally, we also ranked the risk scores of LUAD and LUSC patients for OS and explored the distribution features (Fig. 6C-D). The dot plots revealed the status of each patient in the training sets (Fig. 6E-F). The heat maps showed the differential expression of the feature CCRGs in the high-risk and low-risk groups (Fig. 6G-H). As results show, there was an upregulation of HAL, PHLDB2, AGMAT, DDIT4, CDA, NT5E, and TUBB6 as high-risk genes and downregulation of POU2AF1, SPAG8, and ARRB1 as protective genes of LUAD patients in the high-risk score group comparing the low-risk score group. Moreover, samples with high-risk scores of LUSC patients suggested upregulation of TREM1, WNT10A, CD300C, ALPL, ALOX5AP, VSIG4, RALGAPA2 and PNPLA6 as high-risk genes and downregulation of TIGD3 and HIST1H2BH as protective genes.

### Validation of the prognostic risk-score model in the testing set

**Figure 7:**
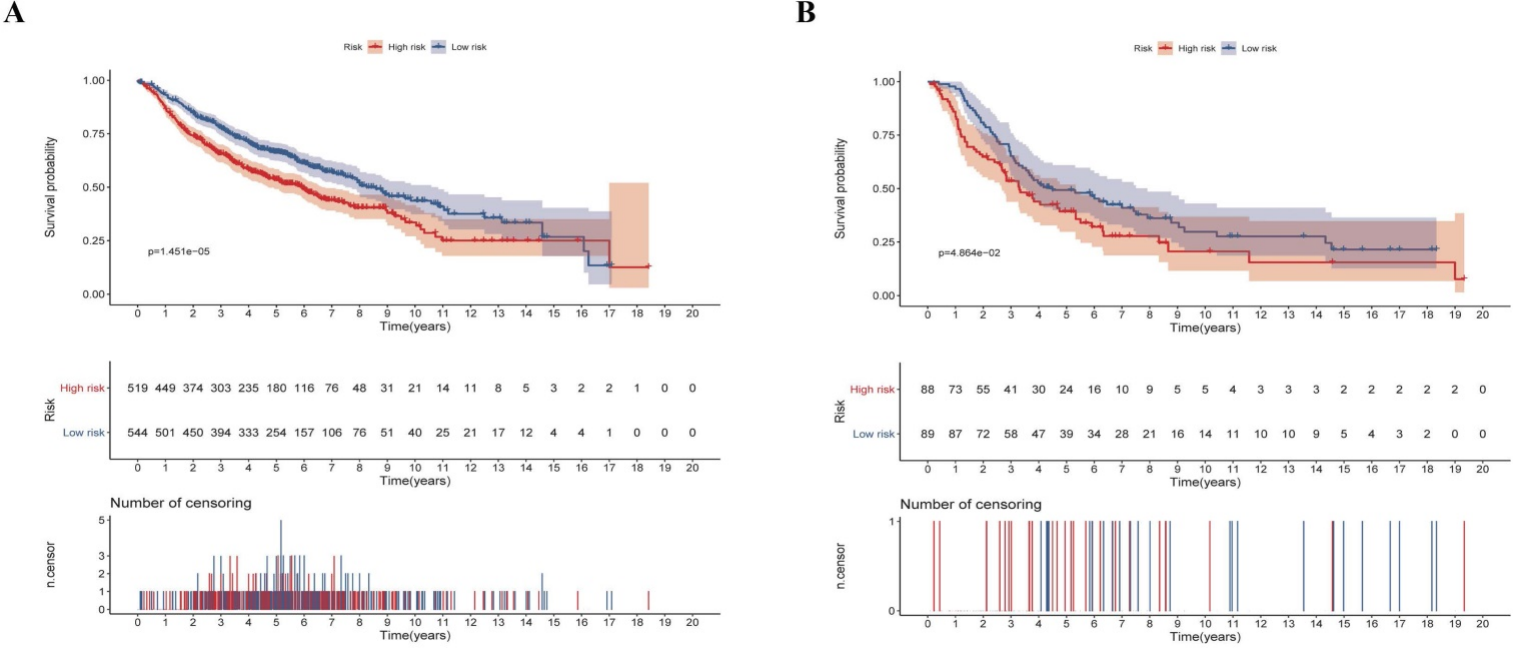
Validation of the risk-score in the testing set. (A) Kaplan-Meier analysis of LUAD patients stratified by the risk-score in the validation set. (B) Kaplan-Meier analysis of LUSC patients stratified by the risk-score in the validation set.

We next validated the stability and accuracy of the prognostic risk-score model in the testing sets, which included LUAD and LUSC cohorts from the GEO database. The OS was selected as the key indicator to compare the groups and samples were divided into low and high risk-score groups based on the calculated risk score. The formula is as mentioned before. For the testing set of LUAD type, 519 and 544 samples were separated into low and high risk-score groups, respectively. Survival analysis showed that there was a significant difference between the high and low risk-score groups (p < 0.0001, HR: 1.493, 95% CI: 1.248 to 1.787) (Fig. 7A). Similarly, 88 samples of the low-risk group and 89 samples of the high-risk group were included in the survival analysis of the LUSC testing set. Results also suggested a significant difference between the high and low risk-score groups (p = 0.0486, HR: 1.453, 95% CI: 1.002 to 2.105) (Fig. 7B). To summarize, our results confirmed that these two risk-score models based on CCRGs signatures were all stable and accurate in predicting the prognosis of patients.

### Clinical characteristics correlation analysis

**Figure 8:**
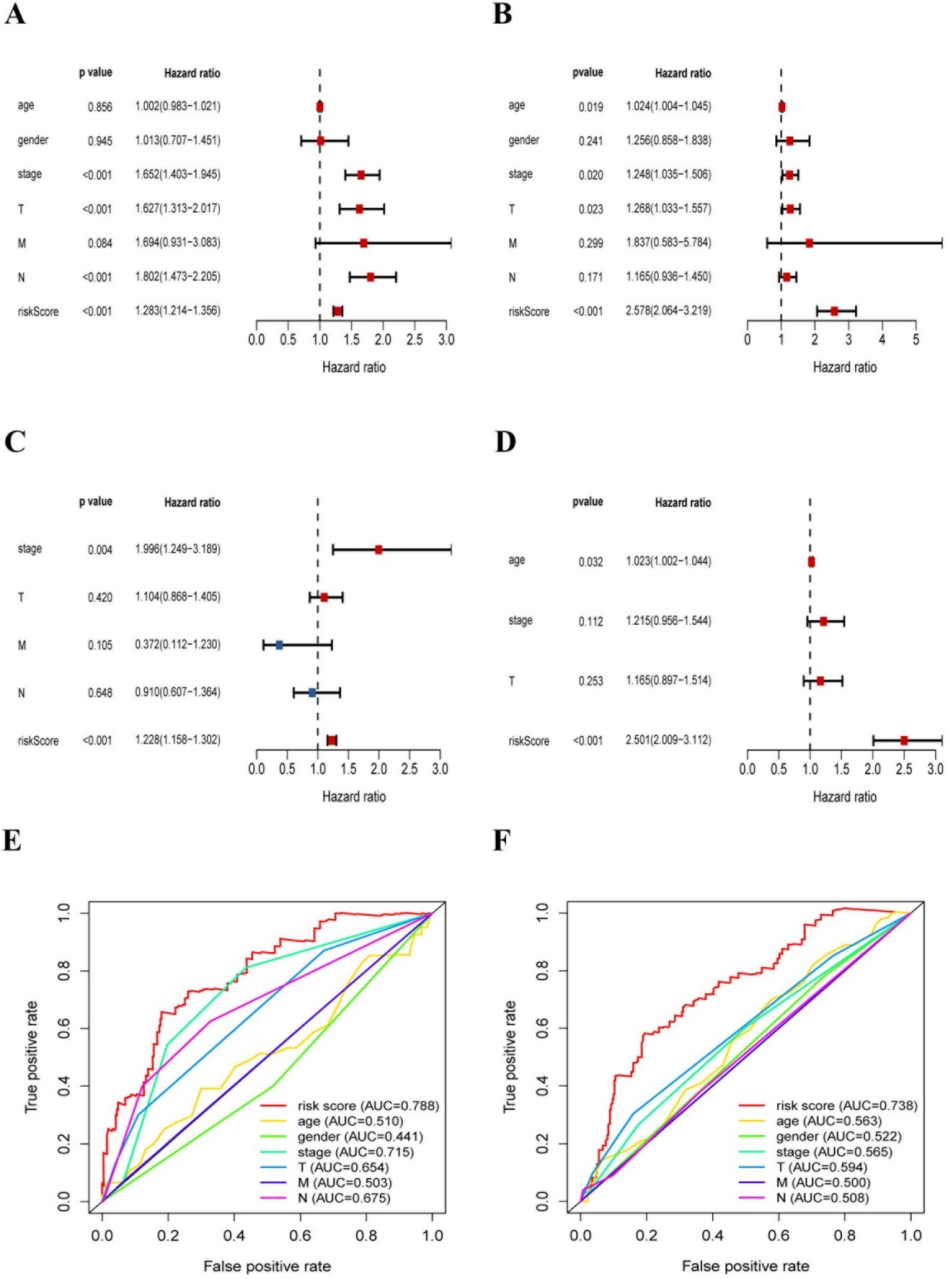
The risk-score was a significant signature with superior accuracy and predictability related to survival in NSCLC. (A) Univariate analysis of clinical characteristics, including the risk-score in LUAD patients. (B) Multivariate Cox regression analysis of clinical characteristics in LUAD patients, the risk-score was an independent predictor of prognosis. (C) Univariate analysis of clinical characteristics, including the risk-score in LUSC patients. (D) Multivariate Cox regression analysis of clinical characteristics in LUSC patients, the risk-score was an independent predictor of prognosis. (E) ROC and AUC analysis of the sensitivity and specificity for the risk score in LUAD patients. (F) ROC and AUC analysis of the sensitivity and specificity for the risk score in LUSC patients.

In this section, we further explored the stability and reliability of the risk score as a clinical indicator. Seven variables, including age, gender, T stage, N stage, M stage, pathologic stage, and risk-score, were analyzed using the univariate analysis and Cox proportional hazard regression in the training sets of LUAD and LUSC respectively (LUAD: p < 0.001, HR: 1.228, 95% CI: 1.158 to 1.302; LUSC: p < 0.001, HR: 2.501, 95% CI: 2.010 to 3.117) (Fig. 8A-D). Our analysis showed that risk-score was found to be an independent prognostic indicator both in LUAD patients and LUSC patients. Then, we constructed ROC curves for different variables to evaluate the risk-score as classifiers, and the AUC was calculated and considered as the basis for evaluation (LUAD: AUC 0.788; LUSC: AUC 0.738) (Fig. 8E-F). Our results indicated that the risk-score had superior accuracy and predictability comparing other clinical characteristics both in LUAD and LUSC samples.

### Immune correlation and infiltration analysis

**Figure 9:**
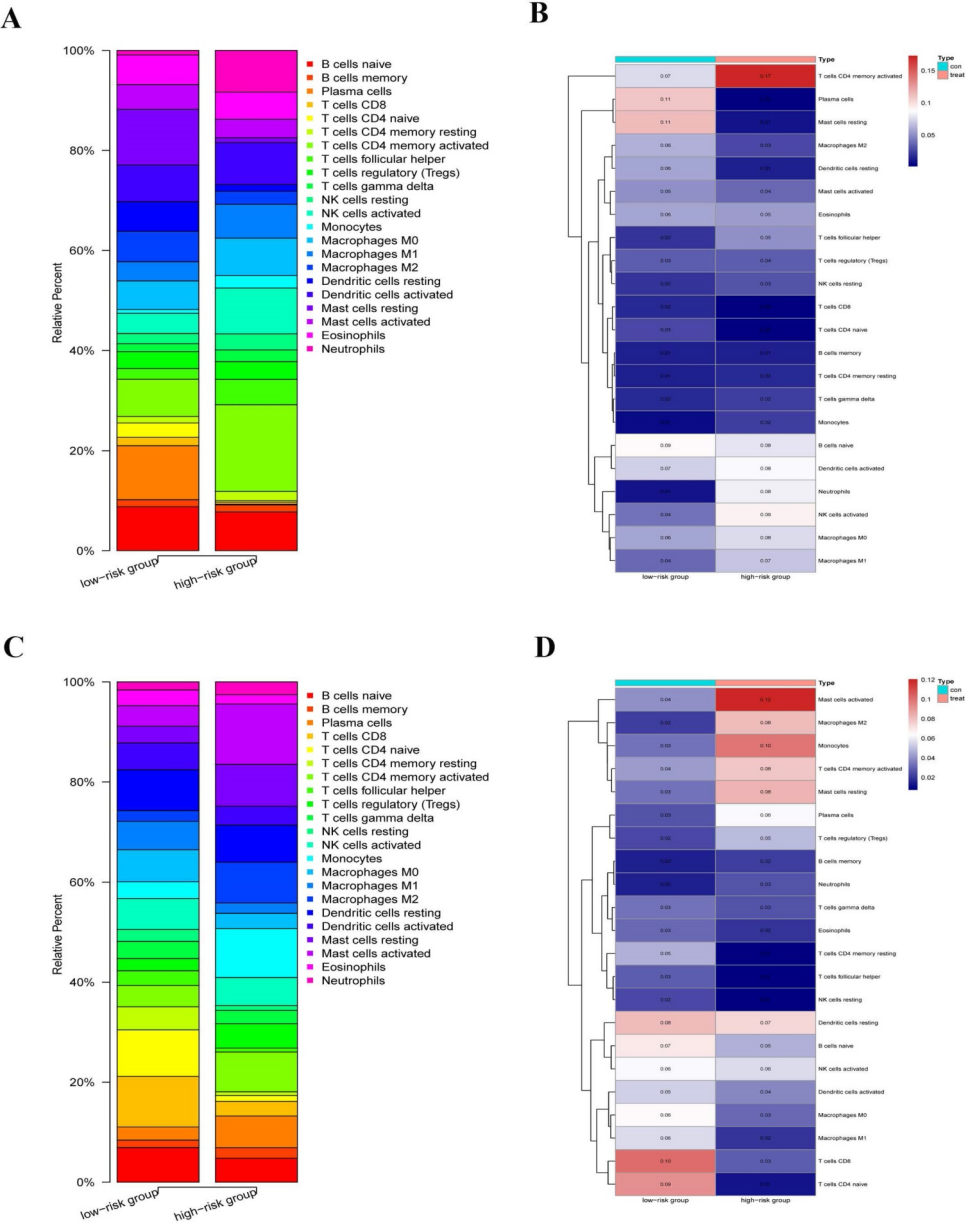
Immune infiltration level of the diverse immune infiltrating cells between the high-risk group and the low-risk group by the CIBERSORTx. (A) Immune infiltration levels of 22 immune cell types in the low-risk group (23 samples) and high-risk group (ten samples) (LUAD). (B) The heatmap of abundances of 22 immune cell types in the low-risk group (23 samples) and high-risk group (ten samples) (LUAD). (C) immune infiltration levels of 22 immune cell types in the low-risk group (19 samples) and high-risk group (26 samples) (LUSC). (D) The heatmap of abundances of 22 immune cell types in the low-risk group (19 samples) and high-risk group (26 samples) (LUSC).

We explored the immune infiltration level of the diverse immune infiltrating cells between the high-risk group and the low-risk group by the CIBERSORTx. We first calculated the abundances of 22 immune cell types in each sample using standardized CCGRs expression profiles in LUAD and LUSC, respectively. Then we filtered each sample according to the p-value to eliminate the bias caused by inaccurate estimation and grouped the samples based on the risk-score. Finally, 23 samples of the low-risk group and ten samples of the high-risk group were selected in LUAD. Meanwhile, of LUSC samples, 19 samples of the low-risk group, and 26 samples of the high-risk group were included for analysis. Our results suggested that immune infiltration levels in the high-risk group of naive B cells, plasma cells, naive CD4+ T cells, CD8+ T cells, dendritic cells, M2 macrophages, and mast cells decreased significantly in the LUAD compared with the low-risk group (Fig. 9A-B). Furthermore, there were significantly increased levels of infiltrates of activated memory CD4+ T cells, T follicular helper cells, regulatory T cells (Tregs), natural killer (NK) cells, monocytes, neutrophils, and M0/1 macrophage in the high-risk group. Moreover, for LUAC samples, immune infiltration levels in the high-risk group of naive B cells, resting memory CD4+ T cells, naive CD4+ T cells, CD8+ T cells, T cells follicular helper, NK cells, dendritic cells, and M0/1 macrophage decreased significantly comparing the low-risk group (Fig. 9C-D). Meanwhile, there were significantly increased levels of infiltrates of plasma cells, activated memory CD4+ T cells, Tregs, mast cells, monocytes, M2 macrophages, and neutrophils in the high-risk group. In summary, we found that there were similar commonalities in the level of immune cell infiltrations between the high-risk group and the low-risk group in LUAD and LUSC. For instance, our results showed that immune cells, which play a vital role in tumor immunity, were significantly decreased in the high-risk group, such as CD8+ T cells, dendritic cells, B cells, and naive CD4+ T cells. Meanwhile, negative regulation and inflammation-related cells, such as Tregs, mast cells, monocytes, and neutrophils, were significantly increased. These results suggested that compared with the low-risk group, anti-tumor immune responses were inhibited while the inflammatory process and hyperresponsiveness may be significantly enhanced in the high-risk group. However, there were still some differences and changes in the level of infiltration of some immune cells between the LUAD and LUSC, which might illustrate the difference between LUAD and LUSC at the level of circadian-rhythm-related immune infiltration.

### Genetic alteration analysis

**Figure 10:**
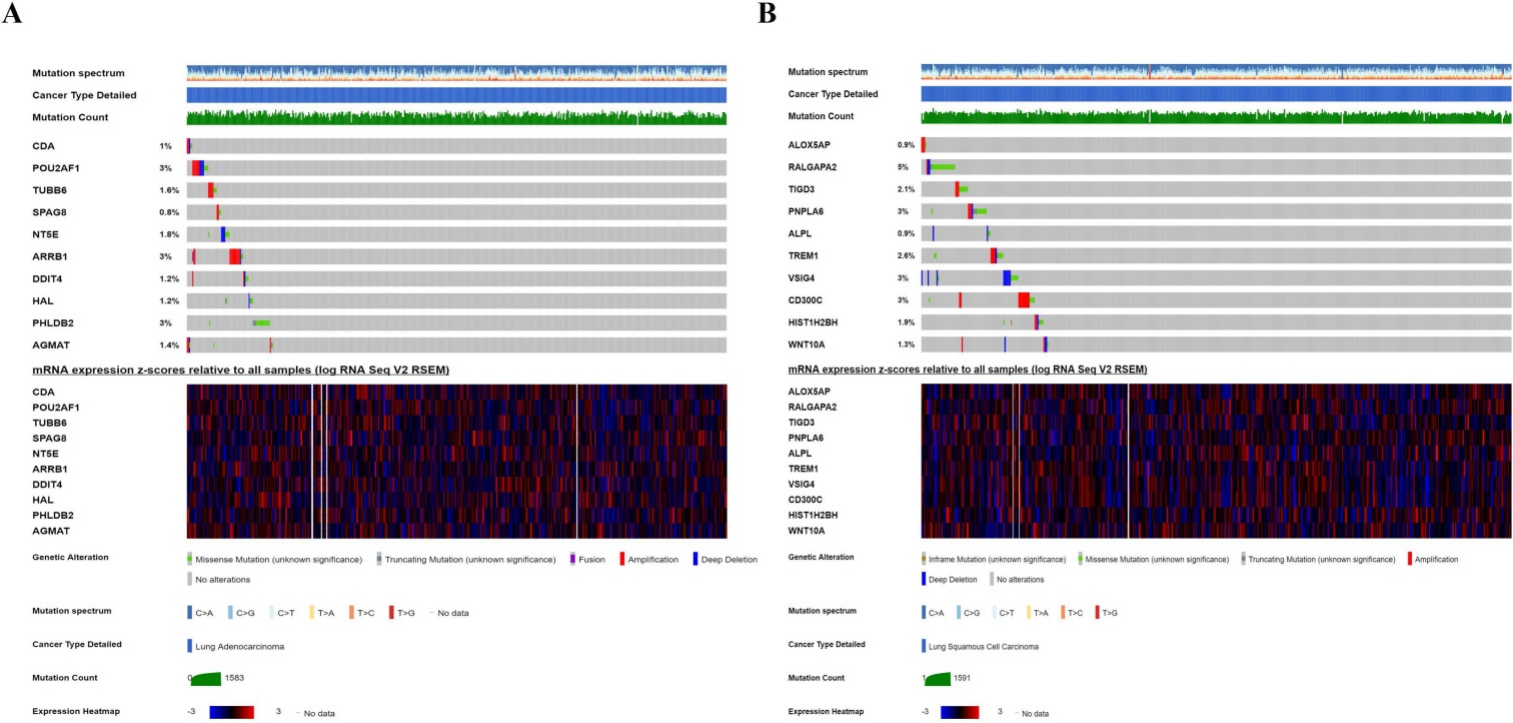
Genetic alteration of ten CCRGs in the risk-score model. (A) For ten CCRGs in the risk-score model of LUAD patients. (B) For ten CCRGs in the risk-score model of LUSC patients.

We investigated the genetic alteration of these CCRGs in the risk-score model to further understand their contributions to carcinogenesis by the cbioportal. We found that these risk-associated CCRGs were relatively conservative, and the mutation rates of these genes were all lower than 3% (most the percent of which were around 1%-2%) both in LUAD and LUSC (Fig. 10). Our results suggested that some of genes with high mutation rate in our risk model may have potential roles in tumorigenesis of NSCLC. The underlying mechanism of those genes with low mutation rate remains to be further investigated.

## Discussion

There is clear evidence for the differential expression of CCRGs in a variety of diseases, and cancers are no exception 24. Also, the process of differential expression was 24-hour periodicity and might also be affected by the seasons. Universally recognized, implications of regulating CCRGs expression in epigenetic control mechanisms have been described during the tumor initiation and progression, which included circadian metabolic changes and tumor-derived macroenvironment, which has been reported in studies of breast cancer and LUAD in mouse models 25. The implication also has been indicated by epidemiological studies 26. Changes and disruptions of circadian rhythms in humans significantly impinged on the increasing risk of tumorigenesis 27,28. Evidently, circadian biology is becoming a critical involvement in improving the understanding of molecular mechanisms involved in cancer cells. Nevertheless, its importance has sparsely been well recognized in clinical studies and practice, and even more when translating to the bedside. Based on this, we attempted to represents a substantial step toward that direction, which aims first to describe landscapes and implications of these differentially expressed CCRGs and investigate the connection between impingement of circadian rhythms and prognostic significance, in the most common and malignant tumor. We finally integrated and analyzed the expression profiles of 1,382 human CCRGs in NSCLC wholly and systematically via the CGDB, TCGA, and GEO database. We pioneering proposed a CCGRs-based risk-score model better to assess the effects of circadian rhythm on prognosis accordingly. In addition, according to the score, we further focused on the infiltration changes of immune cells, genetic alteration, and the possibility of being a pharmacological target in these samples.

Recently, a study based on integration and analysis of data from the TCGA database has investigated the association between 14 kinds of clock genes and prognostic signatures in NSCLC patients, which also showed that differentially expressed clock genes constitute their characteristic asynchronous circadian rhythms 29. To date, thousands of genes and proteins are considered to be related to the circadian rhythms' oscillation. Given the significance of circadian rhythms in lung cancer, it is reasonable to speculate that CCRGs hold excellent promises in prognostic prediction and that a risk score based on multiple-gene signatures derived from dependable algorithms would be more reliable and superior to any single molecules in predicting prognosis of NSCLC. We, therefore, put forward a risk-score model, in which ten-gene signatures were selected and calculated for evaluating the prognostic risk of LUAD and LUSC training sets, and the predictive validity of the risk-score model was validated in several GEO NSCLC cohorts, respectively. Fortunately, the risk scores significantly stratified patient outcomes and immune cell infiltration levels between the high-risk and low-risk groups. Further, the risk-score also showed its excellent stability and accuracy as a classifier in the Cox proportional hazard regression, including risk-score and other clinical variables. It was evident that the high-risk group has an inferior prognosis and a more reduced anti-tumor immune response in our analysis. In the risk-score model containing ten genes of LUAD and LUSC respectively. Many genes have been shown to be a potential diagnostic and therapeutic target in lung cancer. Hui-Er Zhu and his colleagues have indicated that AGMAT (Agmatinase) might drive tumorigenesis via activating MAPK and PI3K/Akt cascades 30. Also, NT5E (CD73) inhibitors are currently being tested in several clinical trials for the treatment of cancer. It has been suggested that NT5E may be linked to both tumorigenesis and EGFR-related drug resistance in NSCLC 31. Furthermore, several genes, such as TREM1 and VSIG4 in our risk model were also shown to have prognostic significance and may prove to be a novel, efficacious strategy for the treatment of NSCLC 32,33. Certainly, functions and roles of some genes have not been fully confirmed, which requires further research to explore in depth.

In this study, we found that these genes are involved in many biological processes, such as cell cycle control, metabolism, immune-modulating, inflammatory reaction, cytoskeletal reorganization, chromatin remodeling, apoptosis in response to DNA damage repair, and protein synthesis and transportation, through systemic functional analysis. We concluded that these above processes in tumor cells might be affected by the circadian rhythm. Several studies have demonstrated that a wide range of core circadian clock components is epigenetically altered, and this perturbation could promote tumorigenesis, progression, and decreased survival in lung cancer, which also suggested an essential position of circadian homeostasis in the tumor-suppressive role 34,35.

Interestingly, we found that the infiltration level of critical cells in specific anti-tumor immunity process, such as CD4+ T cells, CD8+ T cells, and dendritic cells, were suppressed apparently, while the activity and infiltrating of inflammatory cells and Tregs with negative regulatory ability were promoted in the high-risk group. It established that circadian rhythms and related genes played a vital role in the tumor immune and tumor-associated inflammatory response. The latest studies have confirmed our results. To date, current notion suggests that CCRGs express in most immune cells universally and present a circadian oscillation with a fixed rhythm, which performs essential roles in a wide range of immunomodulation process, including the phagocytosis, apoptosis, the synthesis, and release of cytokines, chemokines, and cytolytic factors, the response occurring through pattern recognition receptors 36. Differential expression of CCRGs also plays a vital role in the development and specification of immune cell lineages 37. This view also reflected in our analysis. For instance, immune infiltration level of resting memory CD4+ T cells and naive CD4+ T cells were decreased, while the level of activated memory CD4+ T cells was increased in the high-risk group. Consequently, it is evidence that alterations in circadian rhythms due to differential expression of genes in cancer cells may lead to disturbed the immune responses, and these changes may be caused by clock gene mutation, environmental disruption, or the age and tumor itself. A study of circadian rhythm reprogramming during the lung inflammation suggested that the early events in lung injury may produce a complex reorganization of cellular and molecular circadian rhythms and further regulate immune responses of the host 38. It will be essential to determine the mechanism and causality of oscillations driven by CCRGs in cellular function, metabolism and immunity, and whether the critical drivers for oscillations are the time of day/season-dependence. If so, it might strengthen our fundamental understanding of how the circadian rhythm disturbs metabolism and immune functions to anticipate changes in the environment, and provide a bridge between the circadian rhythms and novel insights to facilitate the development of chronotherapies for fighting cancer and other diseases.

Besides, our genetic alteration analysis also suggested the low mutation rates of these CCRGs in the risk-score model, which was also in line with the current view that CCRGs were evolutionarily conserved in eukaryotes 39. Moreover, this conservatism would affect plenty of critical cell functions, such as immunomodulatory. Studies over the last decade indicate that immune responses related to the circadian oscillators are a consequence of this Darwinian selection process, and the circadian rhythm could minimize costs and maximizes benefits of immunity to optimize organismal fitness in a given environment 40. Thus, the disruption of the normal circadian rhythmic may result in the appearance of CCRGs differential expression and metabolic rhythms, which might function to support host immunity but also increase the probability of tissue damage and a catastrophic vulnerability 41. Meanwhile, we also found the subtle difference between CCRGs differential expression and immune cell infiltration in LUAD and LUSC, which might result from the specific contexts of different types of cancers. These are still urgent questions needed to be studied and solved today.

## Conclusion

All organisms on Earth are exposed to regular environmental cycles generated by the rotation and revolution of the Earth. This, in turn, has led to the evolution of circadian rhythms driven by CCRGs, which facilitate lives to anticipate and adapt to the internal and external changes during their environment. We preliminary explored a risk-score based on ten CCRGs signatures based on TCGA and GEO database in LUAD and LUSC, respectively. This risk-score was an independent predictor of prognosis. Further analysis of cell functions and immune infiltration between the high-risk and low-risk group and genetic alteration of these GGRGs also investigated in our study. Differential expression of CCRGs also regulated the immune cell infiltration level in NSCLC. These CCRGs were evolutionarily conserved with low mutation rates and further studies and experimental confirmations are needed.

## Figures and Tables

**Table 1:** Descriptions of CCRGs of the risk-score model in Cox proportional hazard regression analysis

Type	Gene ID	Full name or Description	Risk coefficient	HR	HR.95L	HR.95H	*p*-value
LUAD	CDA	Cytidine Deaminase	-0.089059134	0.914791	0.822109	1.017923	0.102253
	POU2AF1	POU Class 2 Associating Factor 1	-0.295832916	0.743912	0.631686	0.876075	0.000392
	TUBB6	Tubulin Beta 6 Class V	0.253501154	1.288529	1.031295	1.609925	0.025669
	SPAG8	Sperm Associated Antigen 8	-0.215285427	0.806311	0.602534	1.079006	0.14751
	NT5E	5'-Nucleotidase Ecto	0.165120683	1.179535	1.040611	1.337006	0.009806
	ARRB1	Arrestin Beta 1	-0.229636783	0.794822	0.638692	0.989119	0.039588
	DDIT4	DNA Damage Inducible Transcript 4	0.128789666	1.137451	0.974568	1.327557	0.102412
	HAL	Histidine Ammonia-Lyase	0.254600207	1.289946	1.101163	1.511093	0.001613
	PHLDB2	Pleckstrin Homology Like Domain Family B Member 2	0.232732475	1.262044	1.006844	1.581927	0.043472
	AGMAT	Agmatinase	0.276379132	1.318348	1.00177	1.73497	0.048543
LUSC	ALOX5AP	Arachidonate 5-Lipoxygenase Activating Protein	-0.204079367	0.815398	0.638768	1.040868	0.10134
	RALGAPA2	Ral GTPase Activating Protein Catalytic Subunit Alpha 2	0.209187368	1.232676	0.963034	1.577816	0.096734
	TIGD3	Tigger Transposable Element Derived 3	-0.338879739	0.712568	0.445497	1.139747	0.157327
	PNPLA6	Patatin Like Phospholipase Domain Containing 6	0.262619794	1.300332	0.94122	1.79646	0.11125
	ALPL	Alkaline Phosphatase, Biomineralization Associated	0.088447658	1.092477	0.977686	1.220746	0.118395
	TREM1	Triggering Receptor Expressed on Myeloid Cells 1	0.205116552	1.227668	1.028907	1.464826	0.022834
	VSIG4	V-Set and Immunoglobulin Domain Containing 4	0.21911239	1.244971	1.000762	1.548772	0.049206
	CD300C	CD300 Antigen-Like Family Member C	-0.355141335	0.701074	0.440876	1.114837	0.133452
	HIST1H2BH	histone cluster 1, H2bh	-0.161357251	0.850988	0.749454	0.966278	0.012805
	WNT10A	Wnt Family Member 10A	0.127271005	1.135725	1.008189	1.279394	0.036246

## Abbreviations

NSCLC: non-small cell lung cancer
CCRGs: circadian clock-related genes
LUAD: lung adenocarcinoma
LUSC: lung squamous cell carcinoma
TCGA: The Cancer Genome of Atlas
GEO: Gene Expression Omnibus
GO: Gene Ontology
KEGG: Kyoto Encyclopedia of Genes and Genomes
GSVA: Gene Set Variation Analysis
ROC: receiver operating characteristic
CGDB: Circadian Gene Database
HR: hazard ratio
AUC: area under the ROC curve
BP: biological process
CC: cellular component
Tregs: regulatory T cells
NK cells: natural killer (NK) cells
